# Innovative Technologies for Extraction and Microencapsulation of Bioactives from Plant-Based Food Waste and Their Applications in Functional Food Development

**DOI:** 10.3390/foods10020279

**Published:** 2021-01-30

**Authors:** Monalisha Pattnaik, Pooja Pandey, Gregory J. O. Martin, Hari Niwas Mishra, Muthupandian Ashokkumar

**Affiliations:** 1 Agricultural and Food Engineering Department, Indian Institute of Technology Kharagpur, Kharagpur 721302, West Bengal, India; monalisha.pattnaik21@gmail.com (M.P.); ppandey@student.unimelb.edu.au (P.P.); hnm@agfe.iitkgp.ac.in (H.N.M.); 2 School of Chemistry, The University of Melbourne, Parkville, VIC 3010, Australia; 3 Department of Chemical Engineering, The University of Melbourne, Parkville, VIC 3010, Australia; gjmartin@unimelb.edu.au

**Keywords:** agro-waste, bioactive compounds, therapeutic, encapsulation, functional food

## Abstract

The by-products generated from the processing of fruits and vegetables (F&V) largely are underutilized and discarded as organic waste. These organic wastes that include seeds, pulp, skin, rinds, etc., are potential sources of bioactive compounds that have health imparting benefits. The recovery of bioactive compounds from agro-waste by recycling them to generate functional food products is of increasing interest. However, the sensitivity of these compounds to external factors restricts their utility and bioavailability. In this regard, the current review analyses various emerging technologies for the extraction of bioactives from organic wastes. The review mainly aims to discuss the basic principle of extraction for extraction techniques viz. supercritical fluid extraction, subcritical water extraction, ultrasonic-assisted extraction, microwave-assisted extraction, and pulsed electric field extraction. It provides insights into the strengths of microencapsulation techniques adopted for protecting sensitive compounds. Additionally, it outlines the possible functional food products that could be developed by utilizing components of agricultural by-products. The valorization of wastes can be an effective driver for accomplishing food security goals.

## 1. Introduction 

Fruits and vegetables (F&V) are a significant part of the human diet. Besides containing many nutrients, they are rich in phytochemicals which play a protective role in several chronic diseases [1,2,3]. Nowadays the consumption of F&V has been incorporated into many products such as ready-to-serve beverages, sauces, frozen F&V, fruit juices, nectars, dehydrated pulps, and so on. During the production of these food products, substantial amounts of waste are generated [4]. F&V processing in India, USA, the Philippines, and China produces approximately 1.81, 15.0, 6.53, and 32.0 million tons, respectively, of F&V wastes annually [5]. Major organic by-products from food production include seeds, peels, bracts, leaves, roots, bark, and midribs. These wastes are a potential source for many bioactive compounds (phytochemicals, antioxidants, coloring pigments, and nutrients) having nutritional and functional values. Moreover, proper management of organic by-products can furnish environmental and economic benefits by reducing food loss.

Numerous efforts have been made to utilize bioactive compounds embedded in F&V wastes [6,7]. The bioactive compounds are extracted from the wastes through different extraction techniques [8,9,10,11]. Depending upon the nature of the raw material and the type of bioactive to be extracted, different extraction methods are selected [12]. The recovered bioactive compounds can be used as ingredients to fortify food products, in the pharmaceutical or cosmetics industries [13]. However, there is a high risk of degradation during functional food development. To protect extracted bioactive compounds from severe processing conditions and environmental factors, they can be encapsulated within a coating material [14,15,16,17]. The selection of coating material is dependent on the ratio of the enclosed material (core), which plays a major role in producing uniform spherical microcapsules with high encapsulation efficiency. Contrarily, there are minimal studies that encompass all the fundamental aspects of waste valorization beginning from extraction to functional products via encapsulation. The encapsulated bioactive compounds can be utilized for the development of functional food products that may have many health benefits [18]. These by-products in some forms are added to various food products like meat, sausages, cheese, yogurt, curd, butter, ice-cream, juices, fruit purees, bakery products, and candies [19,20,21].

The present paper provides a comprehensive review of different sources of bioactive compounds generated from F&V wastes and their functional properties. Furthermore, it summarizes some recent advances in the extraction techniques of bioactive compounds. The review paper also explores the effects of operating conditions on the microencapsulation of these compounds. Lastly, it gives a concise overview of the development of functional food products by incorporating microencapsulated compounds.

## 2. Sources of Bioactive Compounds

**Figure 1:**
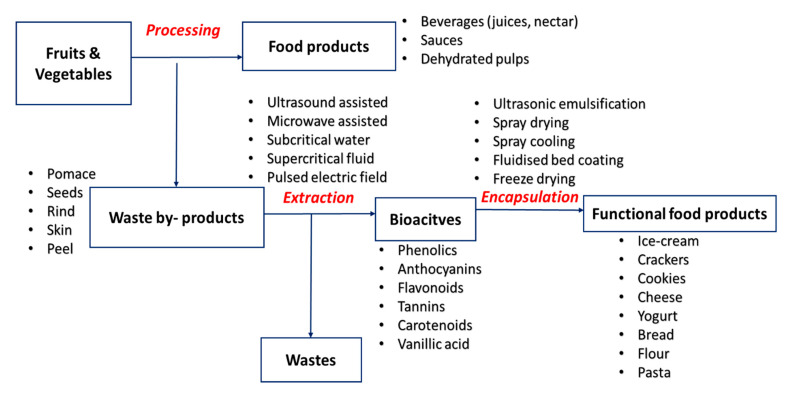
Overview of the utilization of the fruit and vegetables (F&V) by-products from the extraction of bioactive components to food product development.

Food wastes, particularly from fruits and vegetables, are rich sources of bioactive compounds. Bioactive components have elicited nutraceutical effects which are utilized to produce functional foods [8,22]. There have been numerous attempts to recycle these wastes into functional foods to get therapeutic and nutritional benefits. Figure 1 shows a schematic representation of the utilization of food wastes generated from industrial processing. Fruits and vegetables contain bioactive molecules both as primary and secondary metabolites such as lipids, amino acids, fatty acids, polyphenols including hydrolyzable tannins, glycosides, anthocyanin, alkaloids, and flavonoids [23,24]. Antioxidants directly act on quenching of free radicals, slowing down cell damage [25]. Generally, seeds have a pool of polyphenols and other antioxidant compounds while peels are a major resource for dietary fibers [26]. Apart from being an excellent reservoir of bioactive components, the agricultural wastes are also endowed with abundant cellulose, hemicellulose, lignin substances contained in peels, seed coats, or pomace [27,28].

Carotenoids are fat-soluble pigments commonly found in plant tissues that have a good antioxidant activity [29,30]. The predominant forms of carotenoids include lutein, γ, β-carotene, lycopene, zeaxanthin, violaxanthin, antheraxanthin, neoxanthin, and β-cryptoxanthin [31]. There are two classes of carotenoids, (i) xanthophyll which contains oxygen and confers a yellow color; (ii) carotenes that contain no oxygen but only linear hydrocarbons, which can be cyclized at both ends of the molecule, and which confer an orange color. For light absorption in carotenoids, a chromophore group exhibited by conjugated double bonds is responsible. Carotenoids are used in the food industry to replenish color lost due to thermal processing. Islamian and Mehrali [32] suggested that carotenoids have excellent free radical and singlet oxygen quenching capacity. This phenomenon is associated with the inhibition of many free radical influenced diseases namely, atherosclerosis-related cardiovascular diseases [33], multiple sclerosis [34], degenerative diseases [35], and macular degeneration [36]. Graff et al. [37] showed a positive relationship between the consumption of tomato sauce and lycopene and the reduction of prostate cancer. Mezzomo and Ferreira reported a protective action of these compounds for the human immune system along with the enhancement of intracellular communication through gap junctions by second messengers, ions, or metabolites [31]. Carotenes are most bioavailable in their natural trans-form [38,39]. However, isomerization of carotenoids from their trans-form to cis-form occurs in presence of light, heat, metals, or pro-oxidants, resulting in loss of pro-vitamin activity and color [40]. Furthermore, the bioavailability of pro-vitamin A compounds in fruits are greater than in vegetables due to the complex structures of protein in the chloroplast [39]. These compounds after extraction are widely used in the food industry for imparting color (natural colorant), promoting healthy antioxidants, and supplements.

On the other hand, phenolic compounds are characterized by an aromatic ring consisting of one or more hydroxyl substituents. They might involve simple or highly polymerized molecules. There are two classes of compounds; flavonoids and non-flavonoids. The flavonoids encompass subclasses such as flavonols, flavones, flavan-3-ols, anthocyanins, and chalcones while non-flavonoids include stilbenes, phenolic acids (hydroxybenzoic acids and hydroxycinnamic acids), tannins, neolignans, and coumarins [41]. Different phenolic compounds found in peel, pomace, and seeds of fruits and vegetables are summarized in Table 1. Flavonols contain a carbonyl group in their molecular structure. The abundant forms of flavonols found are quercetin and its derivatives, kaempferol 3-O-glucoside, and myricetin. However, phenolic acids with a single phenolic ring are categorized into hydroxycinnamic acids and hydroxybenzoic acids. The hydroxycinnamic acids mainly constitute a three-carbon side chain (C6–C3) in their molecular structure; some of its examples are caffeic, sinapic acids, ferulic, and *p*-coumaric; while the hydroxybenzoic acids group comprises a C6–C1 structure; it covers gallic acid, vanillic acid, *p*-hydroxybenzoic, syringic acids, and protocatechuic [42]. Besides having antioxidant potential, phenolic compounds have received a good amount of attention due to their ability to lower the risk of many chronic diseases such as cancer [43,44], cardiovascular diseases [45,46,47], diabetes [48], neurological disease [49,50], cataract [51], and some disorders of the cognitive function [52,53].

Additionally, the peels of citrus fruits namely lemon, orange, grapes contain distinct compounds likely exo/mesocarp have an excellent amount of flavone and furano derivatives, on the other hand, particularly exocarp is richer in oxygenated monoterpenes [54]. However, a substantial quantity of essential oils is also embedded in the peels (exo and mesocarp), seeds, and other wastes. Lemon essential oils are richer in γ-terpinene and β-pinene, while orange oils have β-myrcene. Grapes and oranges have citral isomers in higher content, whereas lemons contain more valuable essential oils with a greater content of oxygenated compounds [54]. Despite being less explored, the by-products of sapodilla plum (Achras sapota) contain a good amount of saponins and triterpenoids which are widely utilized in folk medicine [55]. These compounds exhibit antimicrobial, spermicidal, anti-inflammatory, and analgesic activities [56,57,58]. Different parts of the plant are inherent with other chemical compounds such as gallic acid, flavonoids, and other phenolic compounds. Interestingly, it has been reported that the introduction of compounds from sapota fruit in the diet can avert the outset of cancer or alleviate the progression of cancer [59].

There are several factors that influence the extraction efficiency of bioactive compounds as well as essential oils, such as the extraction time and technique used, solvent to the amount of sample ratio, and sample matrix (particle size, protein content, oligosaccharide content) [60,61,62,63]. Lafarga et al. [61] reported a reduction in total phenolic content in Brassica vegetables on thermal processing (boiling, steaming), due to the leaching loss of water-soluble phenolics. Contrarily, Su et al. [64] claimed a significant rise in total phenolic content after thermal processing, which can be explained by the rupture of complexes/matrices between phenolic compounds and protein molecules, positively influencing its availability during extraction. Bioactive compounds found in several fruits and vegetable wastes are summarized in Table 1.

## 3. Extraction Methods for Bioactive Compounds

**Figure 2:**
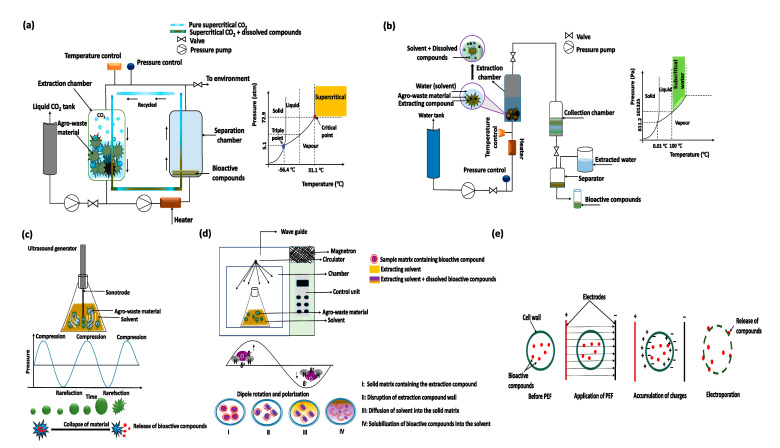
Schematic illustration of extraction techniques, (**a**) supercritical fluid extraction; (**b**) subcritical water extraction; (**c**) ultrasonic assisted extraction; (**d**) microwave assisted extraction; (**e**) pulsed electric field extraction.

There are various techniques for the extraction of bioactive compounds from fruit and vegetable wastes depending on their source, chemical properties, functionality, and end-use. The severity of the extraction methods in terms of temperature, pH, frequency, or electromagnetic waves might have an adverse impact on the extracted compounds. The major extraction methods discussed in this section are listed with key summary information in Table 2. Moreover, the schematic illustration of the extraction techniques are shown in Figure 2.

### 3.1. Supercritical Fluid Extraction

Supercritical fluid extraction (SCFE) using supercritical CO_2_ has been widely used for high-value food applications. Importantly, CO_2_ is non-toxic, better extraction of nonpolar or partially polar compounds in supercritical CO_2_, high solubility of oxygenated organic compounds of medium molecular weight, and non-explosive, in contrast to many organic solvents [160]. It is preferred for the extraction of bioactives from plants or food by-products because of its easy removal from the extracted final product [161].

For extraction, the raw material is initially placed in an extraction container with temperature and pressure controllers, thereafter it is pressurized with the fluid by a pump regulating the temperature conditions. The compounds dissolved in the fluid are conveyed to the separators where the compounds are collected at the bottom and the fluid is either recycled or released to the environment [162]. The critical point of any fluid is marked by its ability to neither behave like gas nor liquid above a critical temperature (CT) and pressure (CP), thus it can easily diffuse into a solid matrix like a gas while also having a high capacity to dissolve compounds like a liquid. Thus, SCFs have an advantage in terms of diffusivity, solute capacity, and low viscosity over other solvents. These characteristics are responsible for better extraction yields and shorter extraction times [163]. CO_2_ with CT 31 °C and CP 74 bar is the most commonly utilized SCF for food application. However, due to its low polarity, the solvation power of supercritical CO_2_ to dissolve the bioactives from a solid matrix gets reduced. Therefore, it is often used in conjunction with a co-solvent or a modifier. Da Porto et al. [164] combined water and ethanol with CO_2_ as co-solvents to extract phenols from grape marc. They indicated that the solubility of the phenols in the supercritical phase reduced at a higher temperature (313.15 to 333.15 K) mainly due to the pre-dominant density effect of SC-CO_2_ + water on the vapor pressure of the extracted compounds, conversely, a predominant effect of vapor pressure over density was observed for SC-CO_2_ + ethanol. However, the extraction yield improved after extracting with SC-CO_2_ + water followed by SC-CO_2_ + ethanol because of the varying polarity of the phenols. The selective extraction of bioactive compounds or co-precipitation of heat-sensitive natural antioxidants can be achieved by micronization through the supercritical anti-solvent (SAS) process where supercritical CO_2_ is used as anti-solvent to precipitate the compounds [165,166].

The steps for extraction of bioactives by SAS process [167,168] are: (1) The solute containing bioactive compounds are dissolved in an organic solvent; (2) CO_2_ continuously flows into the extraction system under a regulated pressure and temperature condition; (3) The solute-organic solvent mixture is then sprayed into SC-CO_2_ where the organic solvent is extracted out of the atomized solute droplet by CO_2_; (4) Due to high miscibility of organic solvent in SC-CO_2_ at super-critical conditions, an instant mutual diffusion occurs at the interface of the solute and SC-CO_2_, this phenomenon induces saturation and phase separation of solute in SC-CO_2_, thus, results in nucleation and precipitation of target bioactive compounds. Zabot and Meireles [169] in their study highlighted the positive effect of the SAS process on the quercetin yield from onion peels. They demonstrated minimum degradation of quercetin because of less exposure to light and oxygen and direct flow of solute organic solvent (ethanol) mixture into the precipitation vessel. According to Czaikoski et al. [170], when propane was utilized as an alternative SCF, both pressure and temperature had a positive impact on the yield. The influence of pressure and temperature on extraction performance vary according to the material type, its origin, and the target compound. For instance, Espinosa-Pardo et al. [171] reported a 24.7% increase in carotenoid yield from peach palm pulp at high pressure and temperature caused by the predominant vapor pressure effect of solute over the density of solvent. The major limitation of SCFE is the slow extraction kinetics [172]. Henceforth, in order to improve the extraction efficiency, it is advisable to couple with other methods like ultrasound or enzyme with SCFE to intensify the mass transfer process by disrupting vegetal matrices.

### 3.2. Subcritical Water Extraction

Subcritical water extraction (SCWE) is another promising environmentally friendly and low toxicity extraction method that can be used as an alternative to traditional techniques. The basic principle of extraction by this technique involves heating water to a temperature between 100–320 °C at a pressure (~20–150 bar). At these given conditions, water remains in its liquid state, however, the dielectric constant of water is altered (i.e., 80 at room temperature to ~27 at 250 °C) [173]. The dielectric constant of water becomes comparable to that of methanol and ethanol which are 33 and 24, respectively at 25 °C. Due to the low dielectric constant of water, the polarity, viscosity, and surface tension are reduced, consequently, the dissolution of non-polar molecules is improved [174]. Based on this unique property, the SCWE method has gained immense popularity for fractionation and extraction of a wide range of compounds with a high degree of specificity. Munir et al. [175] explored the extraction of phenolic compounds from onion skin by employing SCWE for 0.5 h and ethanol extraction for 3 h. They asserted that SCWE produced higher extracts of phenolic compounds than ethanol extraction (200 vs. 70 mg gallic acid equivalent/g) and flavonoids (90 vs. 24 mg quercetin equivalent/g) concentration because of effective disruption of hydrogen bonds, van der Waals forces between analyte and sample matrix and low viscosity of water caused by high temperature and pressure. Similarly, Yan et al. [176] in their studies found that polyphenol extracts of lotus seed epicarp from SCWE exhibited greater radical scavenging ability than hot water extraction (88.72 vs. 30.07 mg gallic acid equivalent/g). To accelerate extraction and to reduce the time of heat-sensitive compounds that are exposed to high temperatures, the raw material can be pre-treated using microwaves, ultra-sonication, or gas hydrolysis (N_2_ or CO_2_). Pre-treatments like microwave and ultrasonication facilitates the diffusion of bioactive compounds into the solvent through sample matrix disruption. On the other hand, N_2_ replaces oxygen in the water and has a shielding effect on the reaction atmosphere that favors the extraction of bioactive compounds [177]. Getachew and Chun [178] reported that amongst all the pre-treatments, microwaves helped in extracting the highest content of bioactive compounds from the spent ground coffee. Some limitations of SCWE include corrosiveness and high reactivity of water at a subcritical state that needs to be considered while designing SCWE equipment [179]. Todd and Baroutian [180] in their study have estimated the cost of manufacture of SCWE unit = NZ$ 89.6/kg product for grape marc.

### 3.3. Ultrasound-Assisted Extraction

Sound at frequencies over 20 kHz, which cannot be detected by humans, is referred to as the ultrasonic region. Ultrasound-assisted extraction (UAE) is one of the promising techniques used for the extraction of bioactive compounds via acoustic cavitation, vibration, and mixing effect generated in liquid media. Generally, the frequency ranges from 20 kHz to 100 kHz and is used for effective extraction of functional compounds from plant materials [181]. UAE efficiency strongly depends upon the physical forces generated by acoustic cavitation, and the 20 kHz to 100 kHz frequency range is known to generate strong physical forces. Acoustic cavitation can result in cell wall destruction which facilitates extraction [182]. The propagation of ultrasound waves in liquid media induces cavitation bubbles to grow and collapse, generating various physical effects that include microjets, shockwaves, and turbulence. These physical forces cause cell wall breakdown, cell surface holes, and exudation of nutrients from the cellular plant matter into the solvent [183]. Additionally, the ultrasonic waves passing through the liquid medium create regions of higher and lower pressure variations known as acoustic pressure. The cavities created by microbubbles on exposure to the acoustic field is dependent on the number of acoustic cycles. The bubble oscillation is accompanied by its expansion during the negative pressure cycle and contraction during the positive pressure cycle [184]. Moreover, the expansion and contraction are followed by the diffusion of vapor in and out of the bubble. This diffusion process causes accumulation of mass in the bubble over time, resulting in net bubble growth known as rectified diffusion. Net bubble growth might also be due to the coalescence of multiple bubbles present in the acoustic sound field. In both ways, the bubbles collapse after growing up to a certain size, which is called resonance or critical size and is inversely related to applied frequency. The low-frequency range (16–100 kHz) is also known as the power ultrasound region where strong physical effects like localized shear and high temperatures occur from the high-intensity collapse of large resonance size bubbles [185]. The cavitation phenomenon intensifies the mass transfer and movement of solvent into the cell matrix. Mostly, water is preferred for UAE, but other solvents namely ethanol, methanol, and hexane are also used. Kaderides et al. [186], observed an increased extraction yield of phenolic compounds from pomegranate peel on increasing the amplitude level up to 40% due to greater contact surface area between the solid matrix and the solvent surface that enhanced the mechanical and cavitation effect of ultrasounds. This increased amplitude caused more violent bubble collapse in the short time since the resonant bubble size is influenced by the amplitude of the ultrasound waves. The generated high-speed jet accelerated the penetration of the solvent into the matrix and the release of phenolic compounds into the solvent by cell wall disruption. González-Centeno et al. [187] found that at 40 kHz, 150 W/L power density and 25 min of extraction time were adequate for extraction of phenolic compounds and flavonols from grape pomace. Cavitation is also influenced by extraction temperature. Sometimes, high temperature improves the solvent diffusion rate by disrupting intermolecular bonds between solvent and matrix. Ahmed et al. [188] investigated the ultrasonic extraction of bioactive compounds from Amaranth extract by varying solution temperature (30–70 °C). They observed the highest phenol and flavonoid contents at 70 °C due to the release of bound polyphenols upon disruption from cell-matrix at a high temperature. Analogous results at 80 °C were also claimed by Das and Eun [189]. Similarly, sample matrix size, state of raw material (powder or leaves), and extraction time were found to influence the overall extraction yield [190,191]. Longer extraction times generated some undesirable changes in the extracted solution, while the small sample matrix size enhanced the contact between the exposed surface and solvent favoring cell pore destruction followed by increased internal diffusion of solute into the solvent. It was found that the particle size of samples varying from 0.54–1.5 mm had the highest oil yield when extracted from date seeds [192]. A comparative study was conducted by Drosou et al. [155] and Safdar et al. [193] utilizing soxhlet extraction and UAE, where UAE ethanol: water (1:1) extracts exhibited the highest phenolic compounds and antiradical activity. Therefore, UAE has an advantage of a shorter time, increased extraction rate, and higher yield over conventional extraction techniques.

### 3.4. Microwave-Assisted Extraction

Microwave-assisted extraction (MAE) is another technique that can be employed in combination with classical solvent extraction. This method is advantageous over traditional extraction methods due to the high extraction rate, less use of solvents, and shorter extraction time [9,194]. The electromagnetic field of microwaves generally ranges from 300 MHz to 300 GHz. Microwave energy is absorbed by the polar materials which are then transformed into heat by ionic conduction and dipole rotation known as dielectric heating. Generally, the solvent with a high dielectric constant is selected for extraction of bioactives from plant matrices for maximum absorption of microwave waves that convert into kinetic energy. The molecules with high kinetic energy thus diffuse into the plant materials resulting in the effective mass transfer of solute into the solvent [195]. However, in certain cases, the plant matrix is directly exposed to microwave heating allowing the solutes to be released into the cold solvent [196,197]. The mechanism of MAE includes 3 basic steps [198]. Firstly, the selective absorption of microwave energy by the water glands inside the sample matrix favors localized heating above or near the boiling point of water causing expansion and rupture of cell walls by disrupting the interaction between the solute and active site of the matrix through the splitting of hydrogen bonds, van der Waals force, and dipole attraction. Second, the disrupted cell promotes the mass transfer of the solvent into the sample matrix and bioactive compounds into the solvent. Third, extracted bioactive compounds then dissolve into the surrounding solvent. Kulkarni and Rathod [154] exploited MAE for extraction of mangiferin from Mangifera indica leaves with water as a solvent. They obtained maximum yield (55 mg/g) at 20:1 solvent to solid ratio and 272 W in 5 min, while Soxhlet extraction produced 57 mg/g in 5 h. The optimum microwave conditions and solvent concentration are the main parameters that vary with the source of raw materials, permeability of the matrix, and the targeted compound. Several researchers extracted bioactive compounds by different extraction techniques to examine their comparative studies on its extraction effectiveness. For instance, Zhang et al. [199] compared a few extraction methods like maceration, percolation, UAE, and MAE for recovery of alkaloids from Macleaya cordata, and MAE had the highest yield of alkaloids (17.10 mg/g sanguinarine, 7.04 mg/g chelerythrine) with the shortest extraction time. MAE is rapid and exploits the advantage of the reduced amount of organic solvent (5 to 10-fold) in contrast to conventional methods with high sample throughput by overcoming the resistance offered by the sample matrix [200]. Conversely, there might be an issue of solute degradation at increased temperatures. Due to the risk of explosions generated by high pressure, special precautions involving the material of construction need to be taken while designing the closed vessel MAE equipment. The industrial scale-up process is achieved with appropriate designing of the reaction vessel, the frequency of electromagnetic radiation, and sample thickness [201].

### 3.5. Pulsed Electric Field Extraction

Pulsed electric field extraction (PEF-E) is an emerging technology used for the extraction of bioactive compounds. It is a non-thermal method that induces cell destruction through the application of electric pulses. These electric pulses are applied in a short duration (usually ranging from milli to nanoseconds) at moderate electric field strength (EFS) [202]. The cells or the sample matrix exposed to these electric fields accumulate charges on either side of the membrane surface, thereby generating transmembrane potential on the cell surface. When the transmembrane potential exceeds a certain critical limit, the weaker sections of the cell membrane create pores otherwise known as cell electroporation [203]. It promotes a substantial increase in permeation across the cell membrane, facilitating the release of intracellular compounds. Therefore, it is known to increase the extraction yield and rates at reduced energy consumption and low environmental impact [204,205]. Furthermore, PEF-E is useful for the effective extraction of heat-sensitive compounds from the sample matrix [206]. As the raw material is placed in between two electrodes inside the treatment chamber, the optimization of the process parameters, including pulse number, electric field strength, treatment temperature, and specific energy input is essential [207]. Fincan et al. [208] subjected beetroots to 270 monopolar rectangular pulses at 10 μs, 1 kV/cm field strength with an energy consumption of 7 kJ/kg for the extraction of betanin. They found that the samples had the highest release about 90% of total betanin in contrast to freezing and mechanical pressing following 1 h aqueous extraction. On comparing with the untreated sample, PEF treated orange peels showed an increase in total phenols from 11.76 to 14.14, 26.92, 29.81, 34.80 mg Gallic acid equivalent/100 g at 1, 3, 5, 7 kV/cm, respectively [209]. Similarly, Delsart et al. [210] reported that moderate electric field treatment and shorter duration (40–100 ms) accelerated the release of phenolic compounds and anthocyanins across the cell membrane. PEF-E showed better selectivity in terms of anthocyanin extraction from grape pomace, other than high voltage electric discharge [211]. This non-thermal treatment can be utilized for selective extraction (temperature <5 °C) by preserving sensitive compounds. PEF-E can also be applied prior to a classical extraction process to reduce the extraction effort [212,213].

## 4. Bulk Encapsulation of Bioactive Compounds

The stability of bioactive compounds is an important criterion to be considered while developing any functional food product. Some health-promoting polyphenols because of their unsaturated bonds in their molecular structure are very sensitive to heat, light, oxygen, and pH [214]. One of the best strategies to protect the sensitive bioactive compounds from environmental impact is by enclosing them in a solid matrix, otherwise known as encapsulation [215]. Encapsulation can also aid in an additional benefit of bioavailability enhancement, masking astringent flavors, and controlled release in the gastrointestinal tract [216]. As there is a variety of possible encapsulation methods, an appropriate technique must be selected based on the target compound and its susceptibility to its operational parameters. Table 3 summarizes the various wall materials used for different bioactive compounds and their suitable encapsulation technique. Moreover, Table 4 describes the in-vivo pharmacological effect and release stability of encapsulated bioactive compounds.

### 4.1. Ultrasound for Bulk Encapsulation

Ultrasound offers a great advantage in the emulsification process for food applications. The prime driving force involves acoustic cavitation where bubbles form, grow and collapse at the emulsion interface resulting in very fine emulsions through disruption and mixing. Two mechanisms are majorly responsible for emulsification; (1) Dispersion of liquid/dispersed phase into second/continuous phase resultant from the interfacial waves produced by sound waves; (2) The acoustic cavitation causes high shear forces that break to the formation of sub-micron sized droplets of liquid phase [185]. The fine-tuning of process conditions such as power density, processing time, and temperature affects the formation and stability of emulsions. It is evident that high intensity and low frequency generate very strong shear forces favorable for sudden bubble collapse dispersing very small droplets of a dispersed phase in the continuous phase, thereby, exceptionally stabilizing the emulsions [240]. Contrarily, Silva et al. [241] observed small lumps in the emulsion (consisting of annatto seed oil and modified starch) due to the gelatinization of starch (wall material) that was promoted by hot spots generated in the emulsion. Hence, for intense process conditions, the cooling of the emulsion during the process is necessary. It is also observed that high shear forces and localized temperature produced during acoustic cavitation have the ability to unfold and denature proteins, while in certain cases it can aggregate proteins through crosslinking (hydrogen bonds, covalent bonds, hydrophobic interactions) [242]. These proteins further aid in the stabilization of the emulsion interface, eliminating the need for surfactants [243]. The formation of emulsions by ultrasonication with dairy proteins as emulsifying agents is of growing interest. The use of ultrasound for the extraction of bioactive compounds is quite popular, however, encapsulation of phenolic compounds is limited [188,189].

### 4.2. Spray Drying for Bulk Encapsulation

Spray drying is most commonly used for encapsulation due to its simplicity, low cost, and ease of scale-up. Briefly, the liquid feed containing a core and coating material is first homogenized into an emulsion. This feed solution is then injected into the drying chamber through an atomizer or nozzle to obtain small microcapsules in the collector chamber after solvent (water) evaporation [244]. Organic solvents are rarely used due to The attributes of spray-dried powders are associated with the operating conditions including feed flow rate, concentration of core and coating agent, speed of atomizer, drying air flow rate, and drying temperature [245]. Generally, polysaccharides (e.g., gum Arabic, maltodextrin, cyclodextrin with varying dextrose equivalent (DE)), and proteins (e.g., whey protein, milk protein, soy protein, and caseinate salts) are used for spray drying [246]. Nogueira et al. [225] demonstrated good retention of antioxidant properties of spray-dried microcapsules of blackberry pulp (coating material arrowroot starch/gum Arabic: 1:1.78). Correia et al. [247] studied the effect of different protein sources (chickpea flour, coconut flour, arrowhead, wheat flour, soy protein isolate) on the encapsulation of blueberry pomace extracts by spray drying. It was evident that micro-particles from soy protein isolate had better storage stability compared to wheat flour, chickpea flour, and coconut flour with the highest antioxidant capacity and showed maximum polyphenols retention (90%) during the storage period. Sormoli and Langrish [248] obtained 95% retention of phenolic contents after encapsulating the orange peel extract in whey protein isolate (WPI) by limiting the outlet air temperature to 80 °C for preventing denaturation of WPI. Contrarily, Agudelo et al. [249] demonstrated a significant reduction (c.a. 42%) of phenolic compounds in spray-dried grape pulp containing gum Arabic and bamboo fiber at 120 °C inlet air temperature. The high temperature during spray drying results in the degradation of encapsulated heat-sensitive compounds such as carotenoids, lycopene, thereby lowers its antioxidant capacity [250]. Additionally, the wall materials are mainly carbohydrates having low glass transition temperature and change their state from glassy to rubbery during spray drying that forms highly sticky powder [251]. Therefore, there might be a chance of solid loss and less product recovery due to the firm sticking of powder on the cyclone separator [252].

### 4.3. Spray Chilling for Bulk Encapsulation of Temperature-Sensitive Bioactives

To avoid the high drying temperature, spray chilling is often employed for encapsulating sensitive bioactive compounds. The basic principle is analogous to spray drying. However, the prime distinction in spray chilling is the replacement of the drying chamber by a cooling chamber, where, as the atomized particles fall into the cooling chamber, their energy is removed for cooling or gelling of droplets. Mostly molten carriers such as hydrogenated vegetable oils or lipids (with melting point 45–122 °C) are used as coating materials [14,253]. Depending on the surface area and size of the particles, the cooling capacity and chamber size is designed. The temperature in the cooling chamber must be regulated below the gelling/melting point of the solid to induce proper solidification of the molten carrier. For example, spray-chilled particles containing cinnamon extracts rich in proanthocyanidin enveloped in vegetable fats (melting point 48 °C) were produced which had an encapsulation efficiency (>87%) possessing spherical shape with variable diameters and some aggregates indicating larger particle sizes [254]. Moreover, the involvement of lipid as a carrier matrix eliminates the need for solvents in dissolving wall materials. Tulini et al. [255] obtained spray-chilled microcapsules loaded with proanthocyanidin-rich cinnamon extract in vegetable fat with outstanding antioxidant activity and controlled release of pro-anthocyanidins in the simulated gastrointestinal tract. Similarly, Oriani et al. [256] produced ginger oleoresin microcapsules (retention >96%) with oleic acid or palm fat as coating materials. They also reported that an increase in the concentration of unsaturated lipid decreased the microcapsule crystallinity that facilitated the diffusion of compounds through the lipid matrix. As the process does not include solvent evaporation, the capsules produced by this technique are non-porous and dense, thus they are resistant to oxygen diffusion and show excellent stability [257]. For instance, Mazzocato et al. [258] investigated the encapsulation of a heat-sensitive micronutrient (cyanocobalamin) and reported encapsulation efficiency of up to 100%. The solid lipid microparticles had a smooth and spherical surface influencing good powder flowability while promoting superior protection (>91.1%) even after 120 days of storage period at 25 °C in the absence of light compared to free one (75.2%).

### 4.4. Fluidised Bed for Additional Coating

The application of the fluidized-bed coating is a promising technique that allows uniform coating of the core material or additional coating of the powder particles to improve the protection of particle surface from environmental stresses such as pH, temperature, oxygen, or light and enhance functionality/bioavailability of the particles [259]. The particles are suspended by an air stream at a predefined temperature and then sprayed by a coating material through an atomizer. The airstream suspends the particles by overturning the gravitational force of these particles that is mainly due to the particle weight, this state is known as the fluidized state. Carrier materials must possess film forming capabilities, adequate viscosity, and thermal stability. A wide range of materials involving starch derivatives, proteins, gums, cellulose, and molten lipid could be employed for this process [14]. The coating materials can either be sprayed at the top or bottom of the device followed by solvent (water) evaporation. The solvent evaporation by heat and mass transfer can be regulated by the water content, airflow rate, spraying rate, humidity of the inlet air, and temperature of the air [14,260]. Generally, the airflow rate is 80% at the center flow in the inner column and 20% at the peripheral that causes the circulation of powder particles [261]. The powder particles to be coated must be dense and spherical with good flowability and narrow size distribution. Spherical shaped particles require fewer coating materials than non-spherical particles of the same shell thickness due to less surface area. Additionally, dense particles will reduce the accumulation of these particles in the filter bags of the fluidized bed machine [261]. The main driving force for drying is the heat transfer between the coating/particle surface and air. The mass transfer is driven by the partial water vapor difference between the particle surface and air and on mass transfer coefficient [262]. Hence, the water content, temperature, and relative humidity of the air play a major role in controlling the drying rate of the coated particles. This technique has a wide application in encapsulating probiotics and vitamins for enhancing its bioavailability by hindering interactions with other compounds (e.g., tannins, phytates) [263]. The development of agglomerated particles is the major limitation of this technique that occurs when the temperature of the particle surface is above the glass transition temperature of the coating material. This results in the coalescence of the wet coating materials that form liquid bridges with the particles through adhesion. These liquid bridges solidify after drying, forming an agglomerated larger particle [264]. However, the phenomenon of uncontrolled agglomeration is influenced by the process parameters viz. initial fluidization velocity, minimum fluidization velocity, feed flowrate (encapsulating materials). For instance, Benelli and Oliveira [265] in their study reported an increase in percentage agglomeration on decreasing the feed flow rate because of the collision between wet particles that strengthened the cohesive forces formed by the liquid bridges. Thus, future research on the use of this method for coating bioactive compounds with minimal agglomeration needs to be addressed. 

### 4.5. Freeze Drying Bulk Encapsulation

Lyophilization, also called freeze-drying or cryodesiccation, is applied for heat-sensitive bioactive compounds because of its low-temperature dehydration process. It is a multi-stage operation that includes pre-freezing of feed emulsion at sub-zero conditions to concentrate the formulation; freezing stage involves cooling the material below its triple point to ensure proper sublimation of ice crystals; primary drying refers to the drying phase where the vacuum pressure is maintained along with the application of enough heat to induce sublimation; secondary drying aims at removal of unfrozen water molecules by increasing the temperature above the primary drying (<0 °C), typically the product temperature is maintained between 20–40 °C, to break hydrogen bond between the bound water and materials [266]. The freeze-dried powders have low moisture content with high powder porosity due to the slow freezing rate and formation of large ice crystals that induce the expansion of matrix structure during the freeze-drying process [267]. In a study conducted by Rezende et al. [268] on encapsulation of bioactive compounds from acerola pulp and residue, the microencapsulation efficiency of freeze-dried microcapsules was found higher than spray-dried powders (gum Arabic + maltodextrin—1:1). The freeze-dried powders had a porous and irregular surface which accelerated the premature release of core materials during the drying process. Despite the porous surface, the freeze-dried powders showed good antioxidant activity comparable to spray-dried powder. Due to the application of vacuum, it is a relatively energy-consuming process that might be reduced by optimizing the freeze-drying cycle to fit more cycles in the life span as well as the batch drying process is time extensive (around 24–48 h). Overall, the initial investment is the limiting factor, while the operational and capital cost of the industrial freeze dryer was recorded to be 702 €/cycle [269].

## 5. Development of Functional and Nutraceutical Food Products

F&V by-products are rich in bioactive components and can be effectively incorporated into food products. In this way, the F&V waste reintroduces into the food chain and mimic the ecological burden. The developed functional food products with bioactives can have antioxidant, antimicrobial, neurotransmitter, anti-diabetic, antifungal, anticancer properties, etc. [18]. These by-products in some forms are added in various food products like in animal products such as beef, chicken, meat, sausages, etc., dairy products, i.e., cheese, yogurt, curd, butter, ice-cream, beverages i.e., orange, apple, carrot juices and in bakery products like cookies, cakes, muffins, etc., and in candies and fruit purees [21,270]. Table 5 summarizes some recent food products developed from extracted bioactives from various F&V by-products. Lipid oxidation is a serious problem in the processing of food products, thus affecting the organoleptic properties and shortening the shelf life of food products. The addition of bioactives in cheese, butter, curd, meat products, and fish products mimic lipid oxidation. Basanta et al. [271] added β-carotene, lutein, tocopherols, and polyphenols extracted from plum pomace in chicken patties to prevent lipid oxidation. Abid et al. [272] extracted lycopene and phenolics from tomato waste and added them to butter. The authors reported that butter enriched with 400 mg of tomato by processing extract/kg of butter has the lowest peroxide values after 60 days of storage at 4 °C and concluded that lycopene and phenolics extended the shelf life of butter while reducing the lipid oxidation. Beverages are a direct way to consume bioactives. Several studies have reported that TPC and AA were significantly improved after the addition of F&V by-products in beverages [273,274,275,276]. Furthermore, to enrich the nutritional value of bakery products, F&V by-products can be added in form of powders or extracts. For instance, Hidalgo, A., Brandolini, A., Čanadanović-Brunet, J., Ćetković, G., and Šaponjac, V. T. J. F. c. [277] incorporated beetroot pomace extracts (PE) and microencapsulated pomace extracts (PME) in the biscuits. PME-enriched biscuits were rich in TPC, AA, and betanin content compared to PE. In another study, wheat flour was partially replaced by grape pomace powder (0–20%) in the preparation of cookies. TPC, TFC, and anthocyanin content in the cookies were increased 2.3, 2, and 12.5-fold respectively, compared to cookies without pomace powder [278]. It can be seen from Table 3 that maximal extraction was achieved using solvent extraction technology for product development. Very few studies have reported the use of non-thermal extraction techniques. Pasqualone et al. [279] extracted phenolic compounds from artichoke extracts using ultrasonication-assisted extraction technology. The extracts were incorporated in fresh pasta and it was found that antioxidant activity and phenolic compounds increased relative to a control pasta. Amofa-Diatuo, Anang, Barba, and Tiwari [280] extracted isothiocyanates (ITC) from cauliflower stems and leaves using sonication. These extracts were incorporated in apple juice and 10% extract addition was found to be acceptable with good sensory properties. PEF and MAE technologies have been used for the extraction of bioactives however, the development of functional food using PEF and MAE continuous extraction technology is still under research [281,282]. Functional food product development using SCFE and PLE methods is also limited to date [283]. A lot of research has been done for the extraction of bioactives, but their application in the food industry is limited. Recently, Souza et al. [284] extracted total flavonols, gallic acid, and caffeine from the black tea using the PLE technique and developed bread. They found that no loses of extracted flavonols during baking of bread at 180 °C for 20 min. The demand for functional and nutraceutical food products enriched with bioactives is increasing continuously [285,286]. Thus, further research on the development of food products using green extraction technologies like UAE, PLE, MAE, PEF is needed. Moreover, these techniques are the best alternative to conventional methods and require less extraction time, chemical requirements, and low-cost process. The selection of extraction methods may influence the extraction efficiencies in different food products. We need to look for the best extraction technique for the development of specific food products.

In nut and shell, developed food products with F&V by-products are rich in bioactives and fibers. The amount of F&V added in the food products depends upon the dosage of bioactives required, the matrix in which they are added, sensory analysis, and consumer acceptability.

## 6. Summary and Future Trends

Food production and processing results in an enormous quantity of waste. These food wastes contain many beneficial bioactive compounds. The utilization of such food wastes by extracting functional compounds will help reduce environmental waste load and add value to the developed functional food product. Different extraction methods have been illustrated, providing an overview of recent trends to maximize yields. The demand for extracting bioactives by green technology with no solvent or minimal use of GRAS classified solvent is increasing. Targeted selection and optimization of an extraction technique for a specific bioactive may enhance the extraction efficiencies. However, owing to the potential toxicity of some organic solvents, solvents such as CO_2_, water, and deep eutectic solvents can be used as alternatives. Advanced extraction methods that do not require any solvents is a future aim. More focus on non-thermal emergent technologies like PEF, or combinations of two or more techniques, could be given to ascertain the potential to obtain higher extraction yields, lower energy consumption, and environmental impact. Development of value-added food products by incorporating these F&V by-products directly and extracted bioactives can improve the nutritional value of food products. The quality of the bioactive components in the developed food products depends upon the processing methods and parameters. Moreover, there are limited studies on the amount of bioactive reaching the targeted site in the human body. So, further research on in vitro studies and animal studies needs to be done to evaluate the health benefits to the consumers.

## 7. Methodology of the Study

This review was focused on several key aspects that combine both theoretical knowledge and potential practical aspects of valorization of plant wastes. A semi-systematic approach was followed to conduct a literature review. The main criterion we chose was the inclusion of the majority of papers published in the last decade on the topics of this review that had a high citation. We used google scholar and web of science databases.

## Figures and Tables

**Table 1:** Different sources of bioactive compounds from plant products and their key functional properties.

Bioactive Compounds	Functionality for Processed Foods	Claimed Health Benefits	Parts	Sources
Lycopene	Antioxidants, food colorant	Radio protectant [65], anti-cancer agent [66], inhibit neurodegenerative diseases [35], promoter of heart health [67]	Peel- 611.10 mg/100 g DW [68]; Pomace- 28.64 mg/100 g DW [69]	Tomato
Polyphenols (gallic, chlorogenic, caffeic, ferulic, syringic, and *p* coumaric acids); and steroidal alkaloids (α-solanine, α-chaconine, aglycone solanidine)	Antioxidants, thickener	Anti-pathogenic [70], anti-inflammatory [71], anti-carcinogenic activities [71,72], neuroprotective activities [73]	Peel- alkaloids 84–2226 mg/kg [74]; polyphenols 32.87 mg/g DW [75]	Potato
Phenols, β-carotene	Antioxidants, pro-vitamin	Anti-inflammatory [76], anti-cancer agent [77], anti-microbial [78]	Peel: β-carotene 20.4 mg GAE/g DW; polyphenols 1371 mg GAE/g DW [79]	Carrot
Chlorophyll, caryophyllene, phellandrene, pheophytin	Antioxidants	Antimicrobial [80], antidiabetic [81]	Peel: chlorophyll 3.46 mg/g, caryophyllene 1.49 mg/g, phellandrene 1.21 mg/g, pheophytin 1.95 mg/g [82]	Cucumber
*p*-hydroxybenzoic acid, *trans*-*p*-coumaric acid, *p*-hydroxybenzaldehyde, caffeic acid	Antioxidants, fiber-rich component	Antimicrobial [83], treatment for diabetes mellitus [84]	Seeds: polyphenols 2.34–6.12 mg GAE/g DW; Shells: polyphenols 7.41–10.69 mg GAE/g DW [85]	Pumpkin
Anthocyanins, cinnamic acid, dihydrochalcones (phloretin), flavan-3-ol (epicatechin), flavonol (quercitin glycosides)	Antioxidant activity (ROS and RNS), food additive (natural alternative to synthetic antioxidants and anti-microbials)	Reduction of oxidative stress and inflammation properties [86,87], modifications of plasma lipids and lipoprotein levels [88,89], and anti-cancer activity [90]	Wastes (pomace, peel)- Anthocyanins 2.83 g/100 g DW; cinnamic acid 1.06 g/100 g DW; phloretic 569 mg/100 g DW; epicatechin 291 mg/100 g DW; flavonol 768 mg/100 g DW [91]	Apple
malvidin-3-O-glucoside, peonidin-3-O-glucoside, gallic acid, *p*-hydroxybenzoic acid, cinnamic acid, vanillic acid, proanthocyanidins, coumaric acid, chlorogenic acid, engeletin, quercetin, astilbin, resveratrol	Antioxidants (ROS/RNS), natural additive	Cardioprotective effect [92], prevention of metabolic syndrome [93], management of diabetes [94], anti-proliferative [95], anti-microbial/bacterial potential [96,97]	Pomace: anthocyanins 1246.85–2092.93 mg/100 g, total phenolic content 3014.55–5101.82 mg GAE/100 g, total flavonoids 1648.28 to 2983.91 mg CE/100 g, Total anthocyanin 1246.85–2092.93 mg/100 g [98]	Grapes
Gallic acid, delphinidin-3,5-diglucoside, cyaniding diglucoside, sinapic acid, α –punicalagin, β –Punicalagin, ellagic acid, hesperidine, quercetrin	Antioxidants, dietary fibers, single-cell protein, industrial enzymes, functional food ingredients, food additives, food lipid stabilizer, and artificial sweetener	Alleviates hypercholesterolemia [99], hyperpigmentation treatment [100], anti-cancer activity [43], dietary supplements	Peels- polyphenols 249.4 mg/g, flavonoids 59.1 mg/g, proanthocyanidins 10.9 mg/g [101]	Pomegranate
Naringin, eriocitrin, hesperidin, narirutin, limonin	Thickening and gelling agent, stabilizer, food additive	Mucoprotective agent [102], anti-carcinogenic [103], cytoprotective effect [104], prevention of neurodegenerative diseases [49]	Peel: Total phenolic content- 1259 mg GAE/100 g (orange), 1812 mg GAE/100 g (lemon), 793 mg GAE/100 g (mandarin) [105]; Total Flavonoids: 4.52 mg CE/100 g lemon peel [106], TF: 2539.82 mg QE/100 g mandarin peel [107]; lemon seeds- limonin 8.95 mg/g DW; Valencia orange seeds- limonin 10 mg/g DW [108]	Citrus fruits
Gallic acid, anthocyanins, ellagic acid, quercetin, tannins, xanthones, mangiferin and its related compounds, kaempferol	Antioxidants, micronutrient, protein-rich food source,	Modulation of diabetes and dyslipidemia [109], heart-protective effects [46,110], anti-cancer [111,112], anti-inflammatory [113]	Peel- Total polyphenolic content: Raw—90 to 110 mg/g, ripe- 55 to 100 mg/g [114]	Mango
Epicatechin-3-gallate, malvidin-3-glucoside, procyanidin B2, gallic acid, procyanidin B4, anthocyanins, naringin, isoscopoletin, coumaric acid	Antioxidant, Food colorant, food additives	Pain reliever & Anti-cancer agent [115,116], tyrosinase inhibitory [117,118], anti-inflammatory [119], immunomodulatory, anti-glycated, anti-diabetics [120], metalloproteinase activity [121]	Seeds: phenolic compounds 80.9 g/kg DW [122], Pericarp: phenolic content 57.8 mg GAE/g DW [123]	Logan/Litchi
Gallocatechin, catecholamine, anthocyanins, delphinidin, cyanide, ferulic acid, cinnamic acid, Epicatechin, Procyanidin	Antioxidant, thickening agent, natural bio-colorant, bio-flavors, source of macro & micro-nutrients	Anticancer [44,124], anti-bacterial [125], lower plasma oxidative stress [126], treatment of diarrhea [127]	Peel: phenolic content 29.2 mg GAE/g DW [128]	Banana
Ferulic acid, *p*-coumaric acid, Caffeic acid, bromelain	Prebiotic, single-cell protein, anti-browning agent, texture improver	Manage hyperlipidemia [129], analgesic and anti-inflammatory effects [130], blood coagulation [131], anticancer agent for malignant peritoneal mesothelioma [132]	Peel: phenolics 222–428 mg GAE/100 g DW [133]	Pineapple
Carpaine, glucotropacolin, benzylisothiocynate, bemzylthiourea, benzylglucosinolate, sitosterol, hentriacontane, papain, caffeic acid, chlorogenic acid, *p*-coumaric acid, ferulic acid, and vanillic acid	Rich in digestive enzymes	Antimalarial [134], Antimicrobial/Antifungal [135], abortifacient [136], wound healing [137], treatment of psoriasis & jaundice [138]	Seeds: total phenolic content 0.31–0.77 mg/g [139], Leaf/peel: total polyphenols 28.61–63.59 mg GAE/g, flavonoids 8.36–23.45 mg CE/g, Proanthocyanidins 3–8.89 mg CE/g [140]	Papaya

GAE: gallic acid equivalent; CE: catechin equivalent; DW: dry weight.

**Table 2:** Different techniques for extraction of bioactive compounds and their operating conditions.

Extraction Techniques	Sources	Compounds	Operating Conditions						Solvent/Co-solvent	Extraction Efficiency, EE (%)/Yield, EY (g/100 g)	References
			Temperature (°C)/MW Power (W)	Pressure (bar)/Flow Rate of Solvent (ml/min)	Frequency (kHz)	Amplitude (%)/Applied Voltage (kV/cm)	Time (min)	Solid: Solvent			
SE	Pomegranate peels	Carotenoids; Punicalagins and ellagic acids	35; 100	-	-	-	-;5	1:5; 1:5	Hexane, Isopropanol; Water	EE:85.7; 80.3 & 19.7	[141,142]
	Pouteria sapota seeds	Oil	70	-	-	-	360	1:7	Hexane	EE:40	[143]
UAE	Tobacco waste (midrib, dust, scrap)	Chlorogenic	50/50	-	37	-	30	1:10	Ethanol-water	EY:0.35	[144]
	*Citrus latifolia* waste	Catechin and diosmin	50/130	-	20	89	12.5	1:50	Ethanol	EE:93, 89	[145]
	Pomegranate peels	Carotenoids	51.5	-	20	40%	30	1:10	Vegetable oil	EE:93.8	[142]
	*Artocarpus heterophyllus* (Jackfruit) peel	Pectin	60, pH 1.6	-	-	-	24	1:15	Water	EE:14.5	[146]
UAE + PLE	Blackberry, blueberry, and grumixama wastes	Anthocyanin	80/580	100	37	-	30	1:18	Ethanol/water	EY:9.62–11.66	[10]
SCFE	Vegetable peel wastes (sweet potato, tomato, apricot, peach)	Carotenoids	59	350/15	-	-	30	1:15.5	CO_2_, Ethanol	EE > 90	[147]
	Apple pomace	Total phenolic content	45	300/33.3	-	-	120	-	CO_2_, Ethanol	EY:5.78	[148]
	Citrus peels and seeds	Carotenoids	41–45	250–300/27	-	-	120	1.5–2.25	CO_2_, seed oil	EY:0.198	[149]
SCWE	Pistachio hulls	Gallotannin & flavonols	110–190	69/4	-	-	30–50	1:25	Water	EE > 96	[150]
	Mandarin peel	Flavonoids	130	30/1000	-	-	15	1:34	Water	EE-96.3	[151]
MAE	Vine prune residues	Total phenolic content	120	-	-	-	5	1:40	Ethanol -water	EY:2.4	[152]
	*Ocimum basilicum*	Polyphenols	-/442	-	-	-	15	1:10	Ethanol	EY:4.3	[153]
	*Mangifera indica* leaves	Mangiferin	-/272	-	-	-	5	1:20	Water	EY:5.5	[154]
	Red grape pomace	Phenolics	50/200	-	-	-	60	1:50	Water-ethanol	EY:23	[155]
	Cabbage leaves	Phenolic content	~50/100	-	-	-	2	1:10	Ethanol	EY:0.86	[156]
PEF + SLE	Potato peel	Steroidal alkaloids	15–23	-	0.01	-/0.75	200 pulses^@^ 3 μs, 60 min	1:5	Methanol	EY:0.158	[157]
PEF + SE	Blueberry press cake	Total phenolics, anthocyannin	23	-	0.01	-/1–5	Pulse width- 1–23 μs, 24 h	1:6	Ethanol	EE: >63, >78	[158]
PEF + UAE	Defatted canola seed cake	Polyphenols	70/200	-	0.03		900 pulses^@^ 20 μs, 20 min	1:10	Ethanol	EY:2.6	[159]

SE: solvent extraction, UAE: ultrasound-assisted extraction, MAE: microwave-assisted extraction, PLE: pressurized liquid extraction, SCFE: supercritical fluid extraction, SCWE: sub-critical water extraction, PEF: pulsed electric field extraction, SLE: solid-liquid extraction.

**Table 3:** Wall materials used for different bioactive compounds and their suitable encapsulation techniques.

Bioactive Compounds	Wall Materials	Advantages of Wall Material	Limitations of Wall Material	Suitable Encapsulation Techniques	References
Lycopene, Citrus reticulata polyphenol extract	Gum Arabic	Good emulsifying capacity, high solubility	Limited protection to oxidation	Spray drying, freeze-drying	[217,218]
Anthocyanin from blackberry by-products, Betanain	Maltodextrin	Low cost, low oxygen permeability, rapid film-forming ability	Poor emulsifying property increase the viscosity	Spray drying	[219,220]
Limonene, Lycopene, betalains	Whey protein isolate	Excellent emulsifying abilities provide good emulsion stability	Limited heat and freeze stability	Freeze drying, Spray drying	[219,221]
Curcumin, Banana peel extracts, β-carotene	Soy protein isolate	Good emulsifying ability, fast film formation	Soluble in alkaline pH	Freeze drying	[222,223,224]
Blackberry pulp	Arrowroot starch and gum arabic	Gelling agent, good emulsifying ability	High viscosity	Spray drying	[225]
Chokeberry anthocyannanis extract	Pectin	Gelling agent and colloidal stabilizer	Forms clumps during dispersion, encapsulation depends greatly on methylation degree	Spray drying	[226]
Lutein	Inulin	Requires low drying temperature for film formation	Sensitive to environmental conditions	Spray drying	[227,228]
Betanins	Xanthan gum	Stabilizes emulsions, protective film against oxidation	High viscosity at low concentration	Spray/freeze drying	[229]
β-carotene	Gum acacia	Stabilizes emulsions	High viscosity	Complex coacervation by sonication	[230,231,232]
Curcumin	Skim milk powder	Good film forming and emulsifying ability	pH-dependent gel swelling behavior	Spray drying	[233]
Lycopene	Whey protein isolate & Gum acacia	Good retention of bioactive compound	Oxidative degradation and mass loss during drying	Complex coacervation, freeze-drying	[234]

**Table 4:** In-vivo studies showing pharmacological effect and release stability of encapsulated bioactive compounds.

Type of Study	Encapsulated Bioactive Compound	Dose	Duration	Results	References
Randomized	Resveratrol	6 mg	35 days	Inhibition of cell growth in tumor	[235]
Randomized cross-over	Curcumin	1 g	3 days	Biotransformation of curcumin are delayed	[236]
Controlled	E. hirta powder	500 mg/kg bw	15 days	Potential antidiabetic activity	[237]
Randomized	Betanin	60 mg/kg bw	28 days	Positive effect on regulating hyperglycemia, hyperlipidemia, and oxidative Stress	[238]
Randomized	astaxanthin	100 mg/kg	72 h	Rate of release and extent of digestion was improved	[239]

**Table 5:** Development of value-added food products from different waste parts of fruits and vegetables.

Raw Material	Waste Part	Extracted/Target Compound	Raw material Processing Method	Value-Added Product	Reported Functional Improvements	References
Apple	Pomace	TPC, TFC, DPPH; TDF	Tray drying	Gluten-free cracker; Ice cream	Rich in antioxidants, dietary fiber, and minerals, specific for coeliac disease patients; dietary fiber-rich products	[287,288]
Tamarind	Seed	β-carotene, TPC, TFC, TAA, TCT	Sun drying	Cookies and mango juice	Natural antioxidants enhance nutraceutical properties	[274]
Banana	Peel	DPPH, ABTS	Solvent Extraction	Orange juice	Increased antioxidant activity	[275,276]
Grapes	Pomace	TPC, DPPH	Freeze-dried;	Yogurt Cheese	Antioxidant properties, anti-inflammatory, anticancer, antimicrobial, and cardiovascular protective properties;	[289,290]
		TPC, DPPH, FRAP	Solid-phase extraction	Bread	Help in prevention of diseases like atherosclerosis, cancer, cardiovascular disease, and type 2 diabetes;	[291]
		TPC, DPPH, ORAC, ICA	Solvent Extraction	Chicken Meat	Antioxidant properties;	[292]
		TPC, TFC, ABTS, FRAP	Solvent Extraction	Cheese	Improved nutritional properties, sensory attributes like friability and adhesiveness	[293]
		TPC, ARP	Solvent Extraction	Fermented milk	Natural antioxidants	[294]
Beetroot	Pomace	TPC, AA, Betalain	Solvent Extraction	Candy	Rich in betalain, antioxidant, and phenolics	[277,295]
		TPC, FRAP, ABTS, Betacyanins	Solvent Extraction with ultrasound	Biscuit	Increased pathogen resistance, anti-inflammatory effect, and antioxidant activities	
Pineapple	Central Axis	TDF	Freeze drying	Cookies	Improved nutritional properties	[296]
Apple	Endocarp					
Melon	Peels					
Raspberry	Pomace	TPC, TAC, RSC, Free EA, ETs	Solvent Extraction and freeze-dried	Fruit Purees	Antioxidant, antimutagenic, anticarcinogenic, antibacterial, and antiviral properties	[297]
Orange	Peel and pulp	TPC, DPPH	Sonication	Carrot juice	Improved functional quality and shelf life	[273]
Artichoke	outer bracts, leaves and stems outer bracts, leaves and stems outer bracts, leaves and stems, Outer bracts, leaves, and stems	TPC, AA	Ultrasound-assisted extraction (UAE)	Pasta	Nutraceutical properties, reduction of cholesterol, antioxidant properties	[279]
Ripe Mango	Peel	TPC, DPPH	Tray drying	Whole Wheat Bread	Rich in antioxidants, help in the prevention of cardiovascular and neurodegenerative diseases, cancers, etc.	[298]
Mango	Seed Kernel	TPC, DPPH	Solvent Extraction	Mango Powder	Natural antibiotic and antifungal properties	[299]
Blueberry and Cranberry	Pomace	TPC, RSA	Solvent Extraction	Mustard	Anticancer, antioxidant, and antimicrobial properties	[300]
Pomegranate	Peel	TPC, DPPH, ABTS	Solvent Extraction	Curd	Increase the anti-oxidative attributes and shelf life of the product	[301,302]
		TPC, FRAP, DPPH	Solvent Extraction and freeze-drying	Cookies	Antioxidant, antimicrobial & nutraceutical properties	
Pineapple	Peel and stems	Bromelain (BR)	Polyelectrolyte precipitation	Flour	Enhance the growth of good bacteria in the human microbiota, high antioxidant activity in human gut	[303]
Passion fruit and Orange	Albedo	TDF	Oven drying	Cake	Reduce cholesterol, and reduce diabetes risks and obesity	[304]
Tomato	Peels and seeds	TPC, RSA, lycopene	Solvent Extraction	Butter	Extended shelf life of butter with antioxidant properties	[272]
Cauliflower	Leaves and stem	Isothiocyanates (ITC), TPC, TAA	Ultrasound-assisted extraction (UAE)	Apple juice beverage	Anticarcinogenic properties	[280]

TPC—total phenolic content; DPPH—2,2-diphenyl-1-picrylhydrazyl; TDF—Total Dietary Fibre; TFC—Total flavonoid content; TAA—Total antioxidant activity; TCT—total condensed tannins; RSA—radical scavenging activity; FRAP—Ferric reducing antioxidant power; ABTS—2 2’-azino-bis(3 ethylbenzothiazoline-6- sulfonic acid); ORAC—oxygen radical absorbance capacity; ARP—Antiradical power; AA—Antioxidant activity; TAC—Total anthocyanin content; EA—ellagic acid; ETs—ellagitannins contents.
